# The Effect of Urban Basic Medical Insurance on Health Service Utilisation in Shaanxi Province, China: A Comparison of Two Schemes

**DOI:** 10.1371/journal.pone.0094909

**Published:** 2014-04-16

**Authors:** Zhongliang Zhou, Zhiying Zhou, Jianmin Gao, Xiaowei Yang, Ju'e Yan, Qinxiang Xue, Gang Chen

**Affiliations:** 1 School of Public Policy and Administration, Xi'an Jiaotong University, Xi'an, China; 2 Institute of Health Administration and Policy, Xi'an Jiaotong University, Xi'an, China; 3 Flinders Health Economics Group, School of Medicine, Flinders University, Adelaide, Australia; Monash University, Australia

## Abstract

**Background:**

Urban population in China is mainly covered by two medical insurance schemes: the Urban Employee Basic Medical Insurance (UEBMI) for urban employees in formal sector and the Urban Resident Basic Medical Insurance (URBMI) for the left urban residents, mainly the unemployed, the elderly and children. This paper studies the effects of UEBMI and URBMI on health services utilisation in Shaanxi Province, Western China.

**Methods:**

Cross-sectional data from the 4^th^ National Health Services Survey - Shaanxi Province was studied. The propensity score matching and the coarsened exact matching methods have been used to estimate the average medical insurance effect on the insured.

**Results:**

Compared to the uninsured, robust results suggest that UEBMI had significantly increased the outpatient health services utilisation in the last two weeks (p<0.10), whilst the significant effect on hospitalisation was evident in the CEM method (p<0.10). The effect of URBMI was limited in that although being insured was associated with higher health services utilisation, compared with the uninsured, none of the improvement was statistically significant (p>0.10). It was also found that compared with the uninsured, basic medical insurance enrollees were more likely to purchase inpatient treatments in lower levels of hospitals, consistent with the incentive of the benefit package design.

**Conclusion:**

Basic Medical insurance schemes have shown a positive but limited effect on increasing health services utilisation in Shaanxi Province. The benefit package design of higher reimbursement rates for lower level hospitals has induced the insured to use medical services in lower level hospitals for inpatient services.

## Introduction

Before China commenced its economic reform from a centralised economy to a market-driven one in the late 1970s, there was near-universal insurance coverage for both rural and urban populations [1], [2]: the Cooperative Medical System (CMS) for the rural population; the Government Insurance Scheme (GIS) for government employees, retirees, disabled veterans, university teachers and students; and the Labor Insurance Scheme (LIS) for company employees, their dependents, and retirees. However, the health care system was not given sufficient attention during the period of economic transition. Without an efficient cost-control mechanism, the over-utilisation of health services and the abuse of free medical care were widespread [3]. The rapid escalation of health care costs in urban China led in part to a fiscal crisis in both the GIS and the LIS; as a result workers were in effect uninsured [4].

The government and enterprises struggled with the immense financial burden of these two systems, driving the pilot experiments of Medical Savings Accounts (MSAs) in 1994, which latterly expanded to a new city-based social health insurance scheme covering the whole country in 1998, named Urban Employee Basic Medical Insurance (UEBMI) [4], [5]. The UEBMI targeted urban employees in formal sector (about half of the urban population according to Yip and Hsiao [2]), leaving the remaining urban residents (such as the dependents of covered workers) not covered. The increasing demand for health care and the concomitant financial burden facing households made the health care issue a major concern in China [6]. The large share of individual expenditure in national health care spending has lowered levels of equitable access to health care services [7]. The lag between the rapidly growing economy and the rigid health care system has posed significant challenges for the sustainability of health care financing and delivery in China [8].

In late 2007, the Chinese State Council decided to launch a new wave of health care reform. Following the creation of UEBMI for urban employees in 1998, and NRCMS designed for rural populations in 2003, the third major public health insurance scheme, URBMI, was finally established for urban residents, mainly the unemployed, the elderly and children (including students) [9]. Given the divergent levels of economic development across China's regions and membership eligibility, the level of financing and benefit packages varies among the provinces and cities [10].

The UEBMI pilots began in Shaanxi Province from 1999, whilst the URBMI was initiated in 2007. By the end of 2012, there were 5,474,900 people enrolled in the UEBMI and 5,713,200 people enrolled in the URBMI, accounting for around 29.2% and 30.4% of the total urban population respectively [11]. The two schemes have some distinguishing features by design. First, the UEBMI enforces compulsory enrollment, while the URBMI adopts voluntary enrollment. This decision was made after weighing the costs and benefits of the two alternative approaches: while mandatory participation would eliminate the risk of adverse selection, voluntary participation could reduce the high administrative costs [12]. Second, UEBMI stipulates both higher financing and reimbursement levels than URBMI. Third, UEBMI includes a Social Pooling Account (SPA) for inpatient care and an individual MSA for outpatient care, whilst the URBMI only has a SPA covering both inpatient treatment and critical (i.e. chronic or fatal disease) outpatient care. These two insurance schemes also share some similarities, in that (1) both schemes are enrolled at the individual level, and (2) both schemes encourage insured people to use lower level hospitals relative to higher level hospitals for inpatient treatments by assigning higher reimbursement rates to the former hospitals. Take the URBMI reimbursement policy set for inpatient treatment at Xi'an City (the capital of Shaanxi Province) for example, the reimbursement rates decreased from 70% to 40% along with the increasing levels of hospitals, from community health services centres to tertiary hospitals. See Table 1 for a brief comparison of two basic medical insurance schemes in Shaanxi Province. A more detailed introduction about a typical UEBMI scheme and URBMI scheme can be found in other literature [9], [12], [13].

**Table 1:** Urban Basic Medical Insurance Schemes in Shaanxi Province, China (Year 2008).

	UEBMI	URBMI
Year of launch	1999	2007
Level of pooling	city/county	city/county
Enrolment unit	individual	individual
Enrolment type	compulsory	voluntary
No. of enrollees (10,000 persons)	432.7	284.6
% of total urban population	27%	18%
Revenue per capita (Yuan)	1345	109
Expense per capita (Yuan)	996	21
Accumulated balance per capita (Yuan)	1153	95
Social Pooling Account (%)	60%	100
Medical Saving Account (%)	40%	N/A
Benefits	inpatient & outpatient care	inpatient & critical (i.e. chronic or fatal disease) outpatient care

Source: China Labour Statistical Yearbook 2009.

UEBMI - Urban Employee Basic Medical Insurance; URBMI - Urban Resident Basic Medical Insurance.

The impact of medical insurance on improving health services utilisation has been well reported in both developing and developed countries [14]–[16]. The empirical evidence regarding urban basic medical insurance in China is still limited and inconclusive. By analysing baseline and first wave post UEBMI reform survey data (conducted in 1994 and 1995, respectively) from Zhenjiang City, Liu *et al*. [17] found statistically significant results that the probability of having outpatient visits increased, whilst the likelihood of hospitalisation, the number of annual total outpatient visits and hospital admissions all decreased. Chen *et al*. [18], using four year (2008–2010) panel data from nine cities, found that URBMI significantly increased both the inpatient and outpatient health services utilisation. Li and Zhang [19] is the only study that investigated both UEBMI and URBMI in the same analysis framework. By using the cross-sectional data from the older population (aged 45 or older) in two provinces (Gansu and Zhejiang) in 2008, they found that UEBMI has increased the likelihood of having outpatient services in Zhejiang Province and the chance of having inpatient services in Gansu Province. The effects of URBMI were all insignificant.

The aim of this study is to investigate the effect of two basic medical insurance schemes on health services utilisation in Shaanxi Province, western China. In addition, this paper contributes to the literature by investigating whether insurance schemes have encouraged the insured to use health services in lower level hospitals for inpatient health services, consistent with the incentives of the benefit package design.

## Methods

### Ethics

The study protocol was reviewed and approved by the Ethics Committee of Xi'an Jiaotong University School of Medicine. The data from this study was drawn from the 4th National Health Services Survey (NHSS) in Shaanxi Province surveyed in 2008. The NHSS was organised and directed by the Center for Health Statistics and Information of the Ministry of Health of China. This study only used the survey data from Shaanxi Province, through the Health Department of Shaanxi Province (www.sxhealth.gov.cn). The data was anonymised when we accessed it and no study subject was directly approached.

### Data source

The data used in this study was drawn from the 4^th^ National Health Services Survey - Shaanxi Province. A multistage stratified cluster random sampling method was used. In the first stage, 44 counties/districts were randomly selected, among which 75 townships/streets were further chosen in the next stage. In the third stage, 105 villages/residents' committees were selected. In total, 2721 households (7948 people) in the urban area and 3239 households (10324 people) in the rural area were interviewed. The survey questionnaire consisted of socio-demographic characteristics, medical insurance status, health status, health services demand, and utilisation. The face-to-face interviews were conducted from June 15 to July 10, 2008. More detailed information about the NHSS can be found in other literature [7], [20].

In this study, urban respondents aged 15 and older were studied. We further excluded a small proportion of respondents who had purchased commercial medical insurance following the literature [10], [18]. This group of respondents might have behaved differently from people who did not have any medical insurance or those who only had one basic medical insurance (since purchasing more than one medical insurance would face an even cheaper price for health services). The final sample consists of 4862 respondents (from 2115 households), among which 2633 (55%) respondents had purchased UEBMI, 780 (16%) respondents had purchased URBMI and 1449 (29%) respondents did not purchase either of the two medical insurance schemes.

### Statistical methods

Denote U_1_/U_0_ as health services utilisations with/without medical insurance (the treatment). In an ideal but counterfactual scenario, we observed both U*_i_*_1_ and U*_i_*_0_ for each individual *i* in a population of interest. The average treatment effect (ATE) for that population is calculated as ATE ≡ E(U_1_−U_0_). However, the ATE isn't the focus for this study since we were more interested in investigating whether purchasing medical insurance could significantly change the health services utilisation behaviours of the insured, i.e. the average treatment effect on the treated (ATT). Let a dummy variable T indicates whether being insured (i.e. T = 1 if insured, T = 0 otherwise), then the ATT could be specified as ATT ≡ E(U_1_−U_0_|T = 1). Since medical insurance status is not randomised in the population, the self-selection issue could not be omitted. In this study, two nonparametric matching techniques were adopted to estimate the average treatment (medical insurance) effect on the treated (the insured). In essense, the matching approach helped to identify the counterparts for the insured, based upon the observable pre-treatment characteristics.

#### Propensity score matching (PSM)

The PSM, proposed by Rosenbaum and Rubin [21], is one widely used approach to handle selection effects by approximating the ideal conditionally-randomised experiment [22], [23]. The detailed steps for conducting PSM analysis have been discussed elsewhere [22]. For this study, the steps could be briefly summarised as follows. Firstly, the logistical regression was adopted to calculate the propensity score for each individual. The dependent variable is a dummy variable indicating an individual's insurance status, whilst the independent variables are observed socio-demographic variables that might influence the individual's demand for medical insurance and/or health services utilisation. Secondly, potential matching algorithms were selected. In this study, the parametric one-to-one matching algorithm was chosen. The third step was to select the samples that are within the region of common support (such that any combination of characteristics in the insured group could also be observed in the uninsured group) into the matching analysis by using the minima and maxima comparison approach. Finally, the performances of matching quality were examined by calculating standard bias and conducting a significance test. Since the propensity score summarised all observed characteristics into one scalar for matching, PSM is referred to as a uni-dimensional matching method.

#### Coarsened exact matching (CEM)

The CEM is an alternative multi-dimensional matching (or monotonic imbalance bounding) method recently proposed by Iacus *et al*. [24]. A key property, opposite to the PSM, is that the CEM bound the maximum imbalance through an ex ante choice specified by the user, i.e. the user would decide the way that observed characteristics were to be coarsened. The user would not need to further conduct balance checking or restrict data to common support as required by the PSM. More beneficial properties and the detailed steps of implementing CEM should be referred to Iacus *et al*. [24] and Blackwell *et al*. [25].

A more general introduction and comparison on PSM and CEM can be found in other literature [26]. The PSM was modeled by using *psmatch2* command coded by Leuven and Sianesi [27]. The CEM was modeled by using *cem* command coded by Blackwell *et al*. [25]. All analyses were performed in Stata version 12.1 (StataCorp LP, College Station, Texas, USA).

### Variables

Four dependent variables were used in this study to evaluate the effect of two urban basic medical insurance schemes on health services utilisation. A binary variable indicating whether an individual had visited a doctor for outpatient treatment in the past two weeks, and a count variable measuring the number of doctor visits in the past two weeks were adopted for the outpatient utilisation. Similarly, a binary variable indicating whether an individual had been hospitalised in the past year, and a count variable documenting the number of hospitalisations in the past year were chosen for inpatient utilisation.

A series of socio-demographic variables which might impact on both the demand of medical insurance and health services utilization were considered in the first step of PSM analysis to calculate the propensity score. The variables chosen were based upon literature review and data availability, including an objective health status indicator (i.e. whether respondents have any doctor-diagnosed chronic diseases), age, gender, marital status, education status, employment status, annual personal expenditure (grouped into three low, medium and high income levels) and sample regions. There was another variable in the survey that could potentially be used to proxy health status – self-reported health status, ranked between 0 (the worst imaginable health status) and 100 (the best imaginable health status). Both of these two proxies have been widely used in the household survey analyses in western China and found to have similar effects [28], [29]. We did not include both indicators in the analysis due to multi-collinearly between two variables and we opted to use the former following the literature [10], [18]. In addition, there were two potential variables that could be used to proxy an individual's income status – the self-reported annual personal income (calculated as annual household income divided by household size) and the self-reported annual personal expenditure (calculated as annual household expenditure divided by household size). As discussed in the literature, respondents in developing countries tended to under-report their income in the household survey, we thus opted to use self-reported annual personal expenditure in the analysis to proxy income [30]. Summary statistics of the survey sample can be found in Table 2.

**Table 2:** Summary statistics for key variables (mean/percent).

	UEBMI	URBMI	Uninsured‡
***Dependent variables***			
Outpatient utilisation			
Any outpatient visit in the past two weeks (%)	7.42	6.69	6.51
No. of outpatient visit in the past two weeks (%)	14.26	12.29	11.26
Inpatient utilisation			
Any hospitalisation in the past year (%)	6.13	4.50	5.32
No. of hospitalisation in the past year (%)	8.03	4.74	6.28
***Independent variables***			
Chronic Disease			
Yes	22.54	18.71	13.27
No†	77.46	81.29	86.73
Age (years)			
15–44†	32.1	60.6	62.0
45–59	41.3	22.9	21.8
>59	26.5	16.4	16.1
Gender			
Male†	54.6	42.1	42.8
Female	45.4	57.9	57.2
Marital status			
Single†	4.6	25.2	26.6
Married	87.6	64.0	63.0
Others	7.8	10.8	10.4
Education status			
Illiterate†	3.1	7.9	8.5
Primary school	8.8	8.3	10.3
Junior middle school	26.1	28.6	32.8
Senior middle school	39.1	42.7	34.2
Diploma	14.3	8.4	7.2
College degree or above	8.5	4.1	7.0
Employment status			
Employed†	50.0	28.0	20.7
Retired	42.1	7.4	7.3
Student	0.1	13.5	16.8
Unemployed	7.8	51.1	55.2
Annual personal expenditure (RMB, 2008)			
Low†	4302(982)	3420(890)	3733(1155)
Middle	7083(885)	5936(757)	6954(868)
High	12533(4099)	10488(3454)	12195(4075)
Region			
Shanbei†	6.3	4.6	8.9
Guanzhong	87.5	95.3	80.6
Shannan	6.2	0.1	10.5
N	2633	780	1449

† Reference levels in the Logistic regression.

‡ The uninsured is defined as those who did not purchase any medical insurance. Standard errors are in the parentheses.

## Results

### Matching performances

In the first step of PSM approach, the logistic regression was used to generate the propensity score. The regression results are presented in Table 3. As can be seen, age, gender, marital status, education status, employment status, income level and sample region were all statistically significant when associated with UEBMI status. Similarly, with URBMI enrollees and uninsured, health status, education status, employment status, income level and sample region were statistically significant.

**Table 3:** Logistic regression results for propensity scores.

	UEBMI		URBMI	
	Odds Ratio	SE	Odds Ratio	SE
Chronic disease	1.012	0.144	1.666***	0.239
Age (years)				
45–59	2.064***	0.259	1.065	0.141
>59	1.669**	0.374	1.359	0.290
Female	0.727***	0.073	1.091	0.108
Marital status				
Married	2.933***	0.512	0.908	0.142
Others	2.091***	0.521	0.920	0.207
Education status				
Primary school	1.049	0.282	1.016	0.248
Junior middle school	2.275***	0.612	1.271	0.302
Senior middle school	4.624***	1.273	1.760**	0.426
Diploma	7.050***	2.201	1.578	0.460
College degree or above	8.378***	2.900	0.871	0.283
Employment				
Retired	3.619***	0.696	0.605**	0.133
Student	0.005***	0.004	0.587***	0.116
Unemployed	0.093***	0.011	0.734**	0.092
Annual personal expenditure				
Middle	1.900***	0.223	1.636***	0.192
High	1.763***	0.228	1.634***	0.200
Region				
Guanzhong	1.648***	0.317	2.595***	0.535
Shannan	1.076	0.277	0.033***	0.033
LR	2129.23		199.82	
N	3837		2125	

Note: *p<0.1, **p<0.05, ***p<0.01.

Based upon the generated propensity scores, the samples that were within the region of common support were identified for the UEBMI and uninsured group (0.008–0.989) and for the URBMI and uninsured group (0.012–0.681). Excluding samples outside the common support region, the sample sizes are 3760 respondents (including 2566 UEBMI enrollees and 1194 uninsured) for studying the UEBMI effect and 1935 respondents (including 750 URBMI enrollees and 1185 uninsured) for studying the URBMI effect. Based on the one-to-one matching algorithm, the final sample sizes for analysing the health services utilisation are presented in the corresponding result tables.

The PSM has improved the comparability between the insured group and the uninsured group. Except for a few characteristics (i.e. age group 45–59, retired, unemployed and high income group), standard bias for all other variables between UEBMI enrollees and the uninsured are lower than 5% after matching. In addition, the *t* test statistics suggest that for all characteristics, the difference between the UEBMI enrollees and the uninsured is statistically insignificant (p>0.10). A similar finding has been observed for the URBMI enrollees and the uninsured, that after matching, the standard bias for the majority of characteristics between the two groups is lower than 5%; there was also no statistical difference on any characteristics between the two groups based on *t* test statistics. After matching, the mean propensity scores for the UEBMI enrollees and the uninsured were 0.645 and 0.644, whilst for the URBMI enrollees and the uninsured, the scores were 0.413 and 0.412; both statistically insignificant. For detailed PSM quality performance tests results please see Tables 4 and 5.

**Table 4:** Comparison of analysis sample characteristics before and after matching (UEBMI and Uninsured).

	Before Matching				After Matching			
	UEBMI	Uninsured	Standardised Bias	P value	UEBMI	Uninsured	Standardised Bias	P value
Chronic disease	22.8	13.5	24.1	<0.001	18.4	17.5	2.3	0.732
Age (years)								
45–59	40.4	21.7	41.3	<0.001	37.6	34.8	6.2	0.375
>59	27.6	16.9	25.8	<0.001	16.6	17.3	−1.6	0.793
Female	45.4	57.5	−24.4	<0.001	50.1	47.3	5.7	0.393
Marital status								
Married	87.5	62.8	59.8	<0.001	81.4	81.0	1	0.867
Others	7.8	10.7	−9.9	0.003	9.1	9.1	0	1.000
Education status								
Primary school	9.3	10.7	−4.8	0.158	10.8	9.3	5	0.445
Junior middle school	25.9	32.0	−13.7	<0.001	29.6	31.1	−3.3	0.617
Senior middle school	39.0	34.0	10.4	0.002	38.0	38.2	−0.4	0.946
Diploma	14.0	7.2	22.3	<0.001	12.3	11.2	3.5	0.611
College degree or above	8.5	7.1	5.1	0.142	5.0	5.4	−1.6	0.767
Employment status								
Retired	43.3	7.5	90.3	<0.001	23.8	20.3	8.7	0.205
Student	0.1	15.8	−60.8	<0.001	0.4	0.4	0	1.000
Unemployed	7.6	56.5	−122.7	<0.001	32.8	30.5	6	0.438
Annual personal expenditure								
Middle	37.2	27.6	20.8	<0.001	34.1	32.2	4.2	0.530
High	37.0	21.9	33.6	<0.001	24.8	29.6	−10.6	0.105
Region								
Guanzhong	88.3	82.3	17	<0.001	85.5	85.3	0.6	0.926
Shannan	5.6	8.7	−11.7	<0.001	7.1	7.3	−0.8	0.899
N	2566	1194			474	474		

**Table 5:** Comparison of analysis sample characteristics before and after matching (URBMI and Uninsured).

	Before Matching				After Matching			
	URBMI	Uninsured	Standardised Bias	P value	URBMI	Uninsured	Standardised Bias	P value
Chronic disease	18.7	13.6	13.9	0.002	14.4	12.4	5.4	0.311
Age (years)								
45–59	23.3	21.7	3.7	0.419	21.7	22.4	−1.6	0.781
>59	16.5	15.8	2.1	0.644	13.7	13.4	0.9	0.867
Female	58.4	56.9	3.0	0.510	56.1	57.6	−3.0	0.601
Marital status								
Married	63.2	61.7	3.2	0.483	62.4	64.6	−4.4	0.438
Others	11.1	10.3	2.6	0.568	9.6	8.6	3.2	0.549
Education status								
Primary school	8.1	9.5	−4.9	0.285	6.8	6.6	0.6	0.909
Junior middle school	28.0	31.8	−8.3	0.071	29.8	27.3	4.9	0.340
Senior middle school	43.1	35.5	15.7	0.001	45.2	46.2	−2.0	0.729
Diploma	8.7	7.3	5.1	0.261	7.0	7.9	−3.7	0.511
College degree or above	4.1	7.1	−13.3	0.005	4.1	4.1	0.0	1.000
Employment								
Retired	7.6	7.8	−0.8	0.865	7.0	6.3	2.5	0.644
Student	13.9	17.5	−9.9	0.032	17.2	16.6	1.8	0.759
Unemployed	51.5	55.0	−6.9	0.130	51.2	52.8	−3.3	0.565
Annual personal expenditure								
Middle	36.6	31.0	11.8	0.009	34.0	36.9	−4.28	0.264
High	36.1	32.4	7.8	0.085	40.9	37.4	3.97	0.305
Region								
Guanzhong	95.5	88.9	24.9	<0.001	97.0	96.9	0.6	0.868
Shannan	0.1	1.2	−13.3	0.007	0.0	0.0	0.0	—
N	750	1185			649	649		

The global imbalance measure L_1_ statistics before and after CEM are reported in Table 6. As can be seen the post matching L_1_ statistics are all close to 0, as opposed to the pre matching L_1_ statistics ranging from 0.016 to 0.741. The final sample sizes are 2231 respondents (including 1580 UEBMI enrollees and 651 uninsured) for studying the UEBMI effect and 1527 respondents (including 639 URBMI enrollees and 888 uninsured) for studying the URBMI effect.

**Table 6:** The L1 measure of imbalance before and after Coarsened Exact Matching.

	UEBMI				URBMI			
	Before Matching		After Matching		Before Matching		After Matching	
	L1	mean	L1	mean	L1	mean	L1	mean
Chronic disease	0.085	0.085	2.80e-16	1.10e-16	0.058	0.058	1.80e-16	−1.40e-16
Age	0.278	0.375	1.70e-16	6.70e-16	0.026	0.036	3.60e-16	−5.10e-15
gender	0.113	−0.113	1.10e-16	−1.10e-16	0.016	0.016	4.40e-16	2.20e-16
Marital status	0.231	0.167	1.50e-16	0.00e+00	0.022	0.028	4.90e-16	−2.00e-15
Education status	0.128	0.340	2.10e-16	4.40e-15	0.089	0.036	7.00e-16	−4.40e-15
Employment	0.638	−1.431	1.20e-16	1.30e-15	0.075	-0.187	8.30e-16	−4.40e-15
Annual personal expenditure	0.237	0.382	8.30e-17	1.30e-15	0.088	0.131	1.90e-16	−4.00e-15
Region	0.050	0.000	1.90e-17	2.20e-16	0.061	0.051	6.80e-17	−2.20e-16
Multivariate L1	0.741		4.624e-16		0.462		6.429e-16	
N	4082		2231		2229		1527	

In summary, after conducting the matching exercises through either PSM or CEM, the insured and the uninsured groups became comparable based on the observed characteristics.

### The effect of UEBMI on health services utilisation

The ATT of UEBMI is presented in Panel A, Table 7. The result suggests that the probability of outpatient visits and outpatient visit rates in the past two weeks have both significantly increased for the UEBMI enrollees, regardless of which matching method was used (p<0.10). Being insured increased the chance of outpatient utilisation by around 78.3% or 122.1% based on CEM or PSM methods, respectively. Being insured in UEBMI also increased the hospitalisation utilisation; however, the difference was statistically insignificant. Further studying where patients received medical treatment, results suggest that UEBMI enrollees were significantly more likely to have outpatient treatment in the clinics (p = 0.02)/community health service center (p = 0.001) based on PSM/CEM methods. For the likelihood of hospitalisation in the past year, the CEM result suggested that the UEBMI enrollees were significantly (p = 0.06) more likely to receive treatment at city level hospitals (see Table 8 for details).

**Table 7:** Effect of basic medical insurance on health services utilisation.

	Propensity Score Matching				Coarsened Exact Matching			
	UEBMI	Uninsured	χ^2^/t statistics	P value	UEBMI	Uninsured	χ^2^/t statistics	P value
**Panel A - Urban Employee Basic Medical Insurance**								
Any outpatient visit in the past 2 weeks	8.44	3.80	8.79	0.003	5.76	3.23	6.21	0.013
Outpatient visit rate in the past 2 weeks	17.93	7.38	2.28	0.023	9.37	5.84	1.80	0.072
Any hospitalisation in the past year	5.49	3.80	1.49	0.222	4.94	3.38	2.61	0.106
Hospitalisation rate in the past year	7.17	4.43	1.42	0.155	5.95	3.84	1.68	0.094
N	474	474			1580	651		
**Panel B - Urban Resident Basic Medical Insurance**								
Any outpatient visit in the past 2 weeks	5.13	3.64	2.24	0.134	5.48	3.83	2.34	0.126
Outpatient visit rate in the past 2 weeks	8.77	5.79	1.42	0.156	10.17	6.76	1.37	0.170
Any hospitalisation in the past year	3.48	2.65	0.86	0.354	3.91	2.70	1.75	0.186
Hospitalisation rate in the past year	3.81	2.98	0.81	0.416	4.07	2.93	1.15	0.252
N	649	649			639	888		

Note: Chi-square test was used to testify the significant difference for any outpatient visit in the past 2 weeks and any hospitalisation in the past year. T-test was used to testify the significant difference for outpatient visit rate in the past 2 weeks and hospitalisation rate in the past year.

**Table 8:** Effect of UEBMI on health services utilisation by the level of hospitals.

Level of Hospitals	Propensity Score Matching				Coarsened Exact Matching			
	UEBMI	Uninsured	χ^2^ statistics	P value	UEBMI	Uninsured	χ^2^ statistics	P value
**Panel A - Any outpatient visit in the past two weeks**								
Clinics	2.95	0.84	5.62	0.018	1.46	1.08	0.50	0.478
Community health service center	1.69	0.84	1.33	0.248	2.78	0.61	10.32	0.001
District level or above	3.80	2.11	2.32	0.128	1.52	1.54	0.01	0.976
Total	8.44	3.80	8.79	0.003	5.76	3.23	6.21	0.013
**Panel B - Any hospitalisation in the past year**								
District level or below	2.95	1.69	1.65	0.199	2.22	1.84	0.31	0.578
City level	1.90	1.05	1.14	0.285	1.84	0.77	3.50	0.061
Provincial/National level	0.42	1.05	1.31	0.253	0.82	0.77	0.02	0.895
Total	5.49	3.80	1.49	0.222	4.94	3.38	2.61	0.106
N	474	474			1580	651		

Note: Chi-square test was used to testify the significant difference.

### The effect of URBMI on health services utilisation

The impact of URBMI is presented in Panel B, Table 7. Results suggest that URBMI has increased both outpatient and inpatient utilisation; however, the difference was insignificant (p>0.10). Further studies into the level of hospitals where patients received medical treatment found that there was some evidence to suggest that compared to the uninsured, URBMI enrollees were significantly more likely to have outpatient treatment in the community health service centers (p = 0.02/0.09 based on PSM/CEM method). On the other hand, for inpatient treatment, being insured in URBMI meant being more likely to have treatments in the city level hospitals (p = 0.09) based on CEM method (see Table 9 for details).

**Table 9:** Effect of URBMI on health services utilisation by the level of hospitals.

Level of Hospitals	Propensity Score Matching				Coarsened Exact Matching			
	URBMI	Uninsured	χ^2^ statistics	P value	URBMI	Uninsured	χ^2^ statistics	P value
**Panel A - Any outpatient visit in the past two weeks**								
Clinics	1.39	1.23	0.06	0.815	1.56	1.46	0.03	0.873
Community health service center	1.85	0.46	5.42	0.020	1.56	0.68	2.83	0.092
District level or above	2.77	2.47	0.11	0.738	2.35	1.69	0.84	0.361
Total	5.13	3.64	2.24	0.134	5.48	3.83	2.34	0.126
**Panel B - Any hospitalisation in the past year**								
District level or below	2.31	1.23	2.14	0.144	2.19	1.46	1.13	0.288
City level	1.08	0.77	0.33	0.567	1.41	0.56	2.92	0.087
Provincial/National level	0.31	0.92	1.31	0.253	0.31	0.68	0.94	0.333
Total	3.48	2.65	0.86	0.354	3.91	2.70	1.75	0.186
N	649	649			639	888		

Note: Chi-square test was used to testify the significant difference.

## Discussion

By using the Shaanxi Province data of the 4^th^ National Health Services Survey, this paper empirically studied the average treatment effect on the treated for the UEBMI and URBMI schemes in Shaanxi Province, western China. Both the PSM and CEM techniques were adopted to handle the potential sample selection issue. Among four health services utilisation measurements (the probability of having any outpatient visit in the past two weeks, the number of outpatient visits in the past two weeks, the probability of receiving inpatient treatment in the past year and the number of hospitalisations in the past year) studied in this paper, robust results suggest that the ATT only limits the UEBMI scheme on outpatient utilisation.

The findings that the UEBMI had significantly increased outpatient health services utilisation but not hospitalisation, and the insignificant effect of URBMI, are consistent with Liu *et al*. [17] and Li and Zhang [19]. The insignificant effect of URBMI is opposite to the findings of Chen *et al*. [18]. Since one of the key aims of expanding the basic medical insurance is to improve the health services access (especially for the inpatient utilisation), the results from this study seems alarming, especially for the more recently established URBMI. Having said that, it should be also noted that firstly, the relatively small sample size could be a reason for the insignificant statistics. The results reported in Tables 7 all show the anticipated sign of the treatment effect that being insured were associated with increased health services utilisations. Secondly, while UEBMI had been implemented in the Shannxi province for around ten years, the URBMI had just been implemented for a year. It is possible that the URBMI enrollees were not so familiar with the scheme. In addition, the cautious attitude towards the insurance funds spending, especially at the initial stage of insurance implementation, may further limit the insurance effect [8].

The inconsistent results reported from this study, Li and Zhang [19] and Chen *et al*. [18] may also highlight the existence of regional heterogeneity with regards to the insurance policy design and impact. The provincial heterogeneity is evidenced in multiple development indicators, such as GDP per capita, life expectancy, average schooling years, to name but a few. As shown in Lin *et al*. [9], the benefit package design is associated with the regional development and in general the benefit package (such as the reimbursement rate) is better off in the wealthier regions. The sample of Chen *et al*. [18] containing 9 cities varied in different development stages and the pooled sample analysis suggests that the impact of URBMI on health services utilisation was significant. The insignificant effect of URBMI in this study may reveal the need to further improve benefit package design in Shaanxi Province.

This paper also contributes to the literature by studying the levels of hospital visits. Since the documented reimbursement rates decrease when the levels of hospitalisation increase, it is expected that the potential incentive would induce insured people to use the health services provided at the lower level of hospitals for inpatient health services. The results of this paper found encouraging evidence that, consistent with the incentive of insurance design, on average respondents who enrolled in UEBMI or URBMI were most likely to receive inpatient treatment at district level or below hospitals, followed by city level hospitals, and the least likely to use provincial/national level hospitals. Compared to the uninsured, the insured were associated with increased likelihood to use inpatient services at lower level hospitals with decreased likelihood of having hospitalisation at provincial/national level hospitals, although only the increased likelihood at city level was significant based on CEM results (p<0.10). As for the outpatients health services, since the UEBMI insured people would use the insurance funds from their MSAs, there was no additional incentive for them to purchase outpatient services from the lower level hospitals. This is supported by this study comparing the medical services utilisation pattern between UEBMI and uninsured, showing there was no particular trend for the outpatient health services utilisation. For the residents who were insured with URBMI, since only the critical outpatient care were covered, they were more likely to visit higher level health care facilities for more complex diseases treatment.

Two caveats are worth mentioning here. First, by using the matching technique this paper increased the comparability between the insured and the uninsured. However, without handling the unobservable heterogeneity (e.g. risk attitude), the results reported in this paper should be explained as association rather than causal effect. This limitation may particularly impact on studying the ATT of the URBMI since its enrollment is voluntary. The unobservable (and thus unmatched) personal and household characteristics may impact on the results presented. It would be ideal to use longitudinal survey data to study the impact of medical insurance on health services utilisation; however, such data is currently unavailable. Second, as also been discussed in Chen et al. [18] there could be a worry that the uninsured might use their family members' medical insurance cards to get reimbursement in the outpatient treatment. If this scenario happens, the ATT of the UEBMI/URBMI on outpatient utilisation reported in this paper may be underestimated, since a proportion of the outpatient utilisation observed in the uninsured would in fact due to the insurance effect of UEBMI/URBMI. The conclusion on the inpatient utilisation will not be impacted since it is unlikely that an insured individual could use other household members' medical insurance card for hospitalisation. Third, as mentioned earlier, the URBMI had just been implemented for one year in our sample, more recent data on the URBMI scheme is warranted to further study the medical insurance effect in Shaanxi Province.
